# Exploiting dietary fibre and the gut microbiota in pelvic radiotherapy patients

**DOI:** 10.1038/s41416-022-01980-7

**Published:** 2022-09-29

**Authors:** Selina E. Eaton, Justyna Kaczmarek, Daanish Mahmood, Anna M. McDiarmid, Alya N. Norarfan, Erin G. Scott, Chee Kin Then, Hailey Y. Tsui, Anne E. Kiltie

**Affiliations:** 1 Medical School, University of Aberdeen, Polwarth Building, Foresterhill, Aberdeen, AB25 2ZD UK; 2 MRC Oxford Institute for Radiation Oncology, Department of Oncology, University of Oxford, Oxford, OX3 7DQ UK; 3 Rowett Institute, University of Aberdeen, Foresterhill, Aberdeen, AB25 2ZD UK

**Keywords:** Cancer, Microbiome

## Abstract

With an ageing population, there is an urgent need to find alternatives to current standard-of-care chemoradiation schedules in the treatment of pelvic malignancies. The gut microbiota may be exploitable, having shown a valuable role in improving patient outcomes in anticancer immunotherapy. These bacteria feed on dietary fibres, which reach the large intestine intact, resulting in the production of beneficial metabolites, including short-chain fatty acids. The gut microbiota can impact radiotherapy (RT) treatment responses and itself be altered by the radiation. Evidence is emerging that manipulation of the gut microbiota by dietary fibre supplementation can improve tumour responses and reduce normal tissue side effects following RT, although data on tumour response are limited to date. Both may be mediated by immune and non-immune effects of gut microbiota and their metabolites. Alternative approaches include use of probiotics and faecal microbiota transplantation (FMT). Current evidence will be reviewed regarding the use of dietary fibre interventions and gut microbiota modification in improving outcomes for pelvic RT patients. However, data regarding baseline (pre-RT) gut microbiota of RT patients and timing of dietary fibre manipulation (before or during RT) is limited, heterogenous and inconclusive, thus more robust clinical studies are required before these strategies can be applied clinically.

## Introduction

The incidence of pelvic malignancies increases in the elderly [1] and is now a significant problem due to the ageing population [2]. The standard treatment of pelvic cancers involves either surgical removal of the tumour or organ preservation using radiotherapy-based treatments. Radiosensitising chemotherapy may be given concurrently to improve patients’ survival, but this can lead to increased severity of adverse effects [3]. In patients over 80 years old, where these adverse effects are poorly tolerated, radiotherapy is often given alone [4, 5], resulting in compromised tumour control. There is therefore an urgent need to develop new approaches to improving radiotherapy outcomes, both in terms of increasing tumour control and alleviating toxicity.

Research into the gut microbiota (defined as the bacteria residing in the gastrointestinal tract) has increased significantly over the past 15 years, with the gut microbiota being of greater importance in health and disease than previously recognised [6, 7]. It can positively influence immune responses involved in the enhancement of anticancer treatment efficacy and in protection against inflammatory processes, including radiotherapy side effects. Differences in gut microbiota composition and diversity have been found in responders and non-responders to chemoradiation [8] and in patients experiencing different levels of toxicities following radiotherapy [9].

**Fig. 1:**
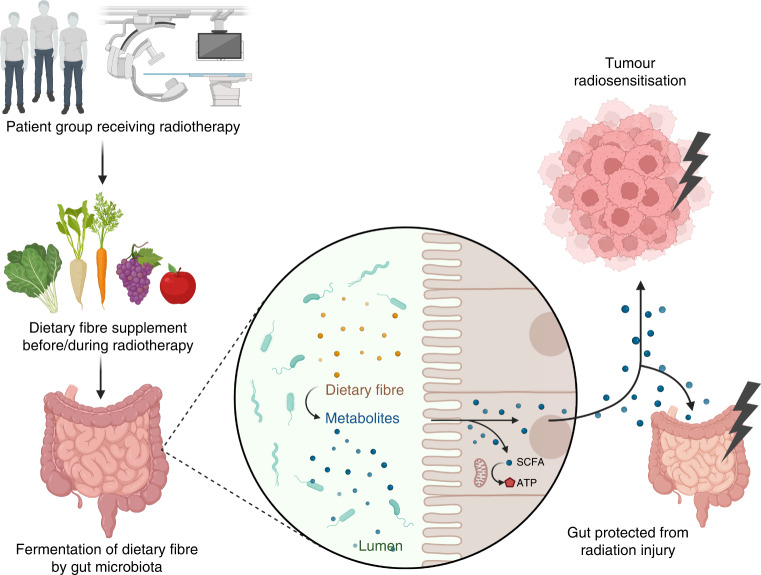
Schematic representation of the proposed effects of dietary fibre manipulation in pelvic radiotherapy patients. Dietary fibre manipulation before or during radiotherapy strengthens or restores the gut microbiota, resulting in an increased production of gut metabolites that may enhance tumour responses and protect the gut from radiation injury. Adapted from “Metabolism of SCFAs”, by BioRender.com (2020). Retrieved from https://app.biorender.com/biorender-templates.

The gut microbiota can feed on dietary fibres which reach the large intestine having avoided digestion earlier in the gastrointestinal tract. Fermentation of the fibre by the bacteria results in the production of metabolites, including short-chain fatty acids (SCFA; including acetate, propionate and butyrate) which are then absorbed by the colonocytes (Fig. 1). A high-fibre diet increases the abundance of specific bacteria that are capable of fermenting these non-digestible carbohydrates, thus increasing SCFA production [10]. Other metabolites have been shown to mitigate radiation-induced intestinal damage, including tryptophan metabolites [11], urolithin A [12] and valeric acid [13].

The gut microbiota interacts with radiotherapy in two ways, impacting treatment response (RT efficacy against tumours [14–16] and normal tissue toxicities [17]) and itself being changed in composition by radiotherapy [18]. Furthermore, altered dietary fibre intake can reduce radiotherapy side effects, leading to a better quality of life for patients [3, 19]. However, while dietary fibre can increase tumour responses to immunotherapy [7] and chemotherapy [20], data on radiotherapy are limited [21].

This review explores the impact of dietary fibre manipulation and the gut microbiota in the treatment of pelvic cancers with radiotherapy and their potential as novel adjuvant therapies to improve patient outcomes.

## Pelvic radiotherapy

Each year, ~12,000 people in the UK receive radiotherapy for their pelvic malignancy [22]. Radiotherapy treatment involves the delivery of beams of ionising radiation to a patient which react with body matter and damage DNA, either directly by breaking down molecules or indirectly by interacting with water in radiolysis to produce free radicals, which if unrepaired can lead to cell death. More recently, the concept of immunogenic cell death (ICD) has emerged, where cell death and alteration of the tumour microenvironment induce an adaptive immune response. This occurs by the release of molecules, collectively termed as damage-associated molecular patterns (DAMP), which stimulate immune cell recruitment. Radiotherapy can initiate this process [23].

The overall aim of radiotherapy is to achieve tumour control (cure) while minimising the acute and late side effects on surrounding normal tissues. Tumour cure requires loss of proliferation in the entire tumour cell population (i.e., sterilisation). The therapeutic ratio aims to balance this tumour cell kill with minimal toxicity to surrounding normal tissue [24]. Tissue responses vary depending on cell turnover rates; both epithelial and hematopoietic tissues have rapid turnover rates, predisposing them to develop acute effects [25]. While combining radiotherapy with chemotherapy may improve tumour control, this may increase side effects.

Modern treatments, for example, 3D-CRT (three-dimensional conformal radiotherapy) and IMRT (intensity modulated therapy), use imaging modalities to precisely outline tumours to reduce the volume of normal tissue in the irradiation field and hence toxicity [26, 27]. IMRT allows the increased dose to the tumour with lower dose deposition in the surrounding tissues compared to 3D-CRT [19, 26]. However, this lower dose “bath” outside of the tumour region may predispose to the development of a second malignancy in these tissues [28], and can increase the volume of intestine irradiated, albeit to a lower dose.

### Pelvic radiation disease

Functional damage resulting from radiation to the abdominopelvic region is now termed “Pelvic Radiation Disease” (PRD) [26] and affects 6000 patients annually in the UK [22]. However, many symptoms (see later) go unreported as the patient assumes them to be typical following treatment.

Radiation enteritis (RE) is characterised by damage to the intestinal mucosa, and the diagnosis is based on the occurrence of PRD symptoms. These symptoms can be described as acute in 90% of patients, occurring during the therapy and up to three months afterwards, or chronic, either as a continuation of acute symptoms or their appearance de novo after three months; up to 90% of patients report a permanent change in their bowel habits and 50% develop symptoms which affect their quality of life. Furthermore, 2–10% develop severe radiation-induced bowel injuries, with 31% requiring surgical intervention [6]. Acute symptoms include diarrhoea, nausea, abdominal pain, fatigue [29], anxiety and depression [30]. In most patients, acute symptoms resolve as intestinal stem cell regeneration recovers the epithelium. In contrast, chronic symptoms are more severe, presenting as fistulae, perforation, abscesses, fibrosis [27] and malabsorption [29]. The risk of developing chronic effects is greater in patients suffering from acute effects [3], although chronic effects can occur alone and, in rare cases, result in intestinal failure [26].

### Cellular effects of radiation in the intestine

Radiation to the abdominopelvic area induces multiple cellular processes, including early disruption of the cell cycle in epithelial intestinal stem cells. These are prone to damage due to their rapid turnover rates, with the small intestine and colon having rates of 1.5 and 4.5 days, respectively [6]. In addition, radiation dramatically impacts the epithelial membrane by compromising the tight junctions. This results in increased permeability, allowing bacteria to invade the surface, thus generating an acute inflammatory immune response [27, 31].

In the later phase, monocytes secrete vasoactive and fibrotic molecules. Myofibroblasts differentiate from fibroblasts, a process modulated by TGF-β1 (profibrogenic cytokine) [32]. A specific increase in the expression of TGF-ß1, collagen, matrix metalloproteinases, macrophages and neutrophils, all of which promote fibrosis, has been found in chronic lesions [6]. The vascular epithelium becomes prothrombotic due to increased expression of cell adhesion proteins and recruitment of leucocytes; if severe, this results in occlusion and ischaemia. Damage involving the enteric nervous system can result in decreased gut motility, encouraging a build-up of Gram-negative bacteria on the villi [31], leading to more inflammation.

## The gut microbiota in health and cancer

The gut microbiota is rapidly colonised from birth, influenced by several factors, including host genotype, lifestyle, diet, environment and the immune system [33]. Changes in dietary habits, use of antibiotics, illness [34] and anticancer treatment [35] can cause chaotic microbiota shifts. The gut microbiota has a huge beneficial impact on human physiological functions, including maintaining mucous membrane integrity, developing normal immune function and conferring protection against enteropathogenic bacteria [36]. However, several species of oral and gut microbiota are associated with cancer development, including gastric cancer (*Helicobacter pylori*) [37], colorectal cancer (*Fusobacterium nucleatum*) [38] and pancreatic cancer (*Porphyromonas gingivalis*) [39]. Reduced abundance of *Clostridium cluster XI* and *Prevotella*, decreased faecal butyrate levels and impaired intestinal integrity was found in newly diagnosed bladder cancer patients [40]. *Parvimonas* was enriched in colorectal cancer [41] and increased with respect to ageing [42] implying that ageing-associated gut microbiota changes could impact cancer development.

### The effects of ageing on the gut microbiota

Pelvic malignancy is prevalent in the elderly population [43]. As age increases, the gut microbiota shifts to a lower diversity [44], with decreased SCFA-producing bacterial abundance [45] and a bacterial composition less likely to respond to anticancer treatments [46]. Three gut microbes, namely, *Akkermansia muciniphila* [47], *Bifidobacterium* [48] and *Faecalibacterium prausnitzii* [49], associated with a better immunotherapy efficacy via immunomodulation and protection against radiation-caused injury, are depleted in older adults [50, 51]. Furthermore, *Bifidobacterium* is depleted in the elderly population [52], and dietary supplementation of probiotic *Bifidobacterium lactis* can successfully restore cellular immune function [53]. Therefore, the gut microbiota could be a therapeutic target to promote tumour responses to radiotherapy in the elderly.

### Immunomodulatory effects of the gut microbiota

The gut microbiota plays a crucial role in multiple immunological processes, including:

1. Maturation and modulation of the immune response via innate immune elements, such as pattern recognition receptors (PRRs) expressed on cells in the intestinal mucosa (e.g., enterocytes, dendritic cells (DCs), macrophages), and adaptive immune elements formed by B cells and T cells [54].
2. Maturation and formation of Peyer’s patches (PP). PP form a part of gut-associated lymphoid tissue (GALT). Specialised microfold cells (M cells) located on their surface sample the antigen and deliver it to antigen-presenting cells within the patches. PP are also sites for B-cell maturation, making them vital for immune surveillance and response.
3. Influencing B-cell activation, either directly via antigen binding to B-cell receptors or indirectly by activation of T cells and innate lymphoid cells, making them essential for maintaining appropriate IgA levels.
4. Inducing increased natural killer (NK) cell activity and secretion of IFN-γ, IL-2 and IL-12 which impacts the activation and phenotype of macrophages, creating an anti-tumour environment. In addition, commensal bacteria can, directly or via DCs, stimulate and prime effector T cells for tumour toxicity.

## The gut microbiota in radiotherapy and pelvic cancers

The interaction of the gut microbiota and cancer treatments is bidirectional, in that these treatments can disrupt the composition of the gut microbiota, and those disruptions can impact on the treatment response and the development of toxicities [55]. The potential mechanisms underlying the bidirectional effects of the gut microbiota and radiotherapy have been extensively reviewed by Liu et al. [56].

### Changes in the gut microbiota pre- and post-IR and its correlation with toxicities

Ionising radiation (IR) significantly alters the gut microbiota profile. The most consistent finding across studies was a significant reduction in richness and α-diversity (within sample diversity, commonly measured by Shannon’s or Simpson’s indices) of the gut microbiota following pelvic radiotherapy [17, 35, 57–59]. A significantly lower α-diversity pre-radiotherapy correlated with the development of toxicities, as seen in patients who developed post-radiation diarrhoea [17]. A relatively lower α-diversity post-radiotherapy was also seen in patients with RE [59] and post-radiation diarrhoea [9, 60]. Patients harbouring a higher α-diversity at baseline had more favourable outcomes, e.g., no self-reported symptoms following radiotherapy [9]. Specific changes in bacterial composition were also reported, although the findings remain inconsistent. Overall, the consensus observation is an increase in phyla Proteobacteria, Fusobacteria and unclassified bacteria and a decrease in phyla Firmicutes and Bacteroidetes and genera *Faecalibacterium* and *Bifidobacterium* following pelvic radiotherapy [61, 62].

Characteristic pre-radiotherapy microbial compositions were seen in patients who later developed RE during pelvic irradiation [59] or post-radiation diarrhoea [17] and in patients demonstrating improved survival [8]. An increased relative abundance of Proteobacteria and decreased relative abundance of Clostridiales and Bacteroides have been reported in patients with non-favourable GI outcomes, such as RE, diarrhoea and colonic fibrosis (Table 1). Proteobacteria are Gram-negative, and the overgrowth of Gram-negative bacteria was vital to the pathogenesis of RE [63]. In contrast, Clostridiales and Bacteroides are SCFA-producing bacteria known to promote intestinal homoeostasis; therefore, their depletion might have detrimental effects (see later). These findings suggest that an increase in deleterious bacteria and a decrease in favourable bacteria may contribute to the development of toxicities, hence recolonising the gut microbiota with favourable species and increasing the diversity pre- and post-radiotherapy may alleviate radiation-induced toxicities.

**Table 1:** Bacteria featured in studies evaluating the association between microbial composition and radiation toxicities.

Model	Treatment	Cancer	Pre-/post-RT and main findings	Reference
Human (*N* = 35)	CRT (Pelvic radiotherapy with cisplatin)	Cervical cancer	Pre-RT. Patients who later experienced a greater decline in GI function had decreased o_Clostridiales and g_*Desulfovibrio*, and increased g_*Sutterella*, g_*Finegoldia* and f_Peptococcaceae	[35]
Human (RE = 10, non-RE = 8)	Pelvic RT (50.4 Gy/28)	Cervical cancer	Pre-RT. Patients who later developed RE had an increased abundance of g_*Coprococcu*s. Post-RT. Patients with RE had a significantly increased p_Proteobacteria, c_Gammaproteobacteria, g_*Serratia*, g_*Bacteroides* and g_*Prevotella_9*, and decreased g_*Bacteroides*.	[59]
Human (acute = 32, late = 87, RE = 9, control = 6)	Prostate and seminal vesicles and/or lymph node RT (55–74 Gy)	Prostate cancer	Pre-RT. Patients with RE had an increased abundance of g_*Clostridium IV*, g_*Roseburia*, and g_*Phascolarctobacterium*.	[90]
Human (cancer = 11, control = 4)	Pelvic RT (44–50 Gy)	Cervical, anal, colorectal cancer	Pre-RT. Patients who later developed diarrhoea had an increased p_Firmicutes to p_Bacteroidetes ratio, an increased abundance of g_*Bacteroides*, g_*Dialister* and g_*Veillonella* and a decreased abundance of g_*Clostridium* XI and XVIII, g_*Faecalibacterium*, g_*Oscillibacter*, g_*Parabacteroides*, g_*Prevotella* and unclassified (genus: others).	[17]
Human (cancer = 10, control = 5)	Pelvic RT (43–50 Gy)	Abdominal tumour	Pre-RT. Patients who later developed diarrhoea had an increased abundance of c_Bacilli and p_Actinobacteria.	[60]
Mouse	Abdominal IR (15 Gy)	–	Post-RT. Transplantation of inulin-derived gut microbiota and metabolites mitigated IR-induced colonic fibrosis. The proportion of the SCFA-producers was significantly higher in the inulin + IR group compared to that found in the IR group. The SCFA-producers include *g_Allobaculum, g_Bacteroides, g_Odoribacter, g_Alloprevotella, g_Parasutterella, g_unidentified_Lachnospiracea, g_Bifidobacterium, g_unidentified_Clostridiales, g_Blautia, g_Intestinimonas*.	[135]
Mouse	Abdominal IR (10 Gy)	–	Pre-RT. Antibiotic cocktail (metronidazole, vancomycin, ampicillin and gentamicin) mitigated radiation-induced intestinal injury by reducing inflammation and intestinal fibrosis. Alpha diversity of antibiotic cocktail group was significantly lower compared to control.	[136]
Mouse	Total body IR (8.0 to 9.2 Gy)	–	Post-RT. Increased abundance of f_Lachnospiraceae and f_Enterococcaceae in the gut microbiome of mice surviving high dose IR (radioprotective effects).	[11]
Mouse	Localised internal RT (22 Gy)	–	Pre-RT. GF mice colonised with irradiated microbiota manifested a more severe damage following irradiation compared to control, and they had increased abundance of p_Proteobacteria, p_Bacteroidetes, g_*Parabacteroides* and *g_Sutterella spp*., and decreased abundance of p_Firmicutes and members belonging to o_Clostridiales.	[65]
Mouse	Total body IR (6.5 Gy)	–	Post-RT. Faecal microbiota transplantation from healthy mouse donor rescued the dramatic decrease of small intestine intact villi and thickened the gut mucosal injured by irradiation. IR reduced g_*Bacteroides* (or g_*Lactobacillus*) and this was restored by faecal microbiota transplantation.	[130]

*GI* gastrointestinal, *Gy* Gray, *IR* irradiation, *N* sample size, *RE* radiation enteritis, *RT* radiotherapy, *p_* phylum, *c_* class, *o_* order, *f_* family, *g_* genus, *s_*species.

The underlying mechanisms of the gut microbiota on IR-caused intestinal injury remain to be elucidated. Germ-free (GF) mice, devoid of the gut microbiota, are remarkably resistant to lethal RE. Histological analysis following 16 Gy total body irradiation (TBI) revealed that GF mice had no evidence of RE, whereas control mice harbouring a gut microbiota displayed an injury response [64]. The colonisation of GF mice with irradiated microbiota and subsequent radiation exposure resulted in more severe tissue damage than in GF mice with naive microbiota [65], and the irradiated microbiota induced pro-inflammatory cytokines, particularly IL-1β, in vitro and in vivo. Antagonising the IL-1 receptor ameliorated tissue damage, implying that IL-1 was a significant driver in its pathogenesis. Therefore, IR-induced alterations to the gut microbiota could themselves contribute to intestinal inflammation and hence exacerbate RE.

As current evidence is mostly limited to association studies between the gut microbiota and normal tissue toxicities, studies involving manipulation of the gut microbiota before and during RT are required.

### Effects of gut microbiota composition on tumour outcome

There has been recent interest in exploiting the effect of the baseline gut microbiota profile on tumour response following immunotherapy [66–68], chemotherapy [69–72], chemoradiation [8] and radiotherapy [14–16], in both preclinical models and human studies. Patients harbouring a higher α-diversity at baseline had more favourable outcomes, as seen in patients with improved tumour responses to chemoradiation, thus leading to improved patient survival [8]. Rectal cancer responders to concurrent chemoradiation had a higher pre-RT abundance of Bacteroidales (Bacteroidaceae, Rikenellaceae, Bacteroides) compared to non-responders [73].

Unfortunately, with high interindividual variability, heterogeneity among studies in humans and mice, and inconsistent findings reported, a specific favourable microbiota profile associated with the development of radiation-induced toxicities (Table 1) and/or tumour response (Table 2) has not been defined. If such profiles were to be established, modulating the gut microbiota by means of probiotics, prebiotics or faecal microbiota transplant could reduce toxicity and improve tumour responses in pelvic radiotherapy patients. Since most studies utilised 16 S rRNA gene sequencing for microbial analysis, which only identifies bacteria at higher taxonomic levels, the need for strain-level metagenomic analysis is therefore important for application in clinical practice.

**Table 2:** Bacteria featured in studies evaluating the association between microbial composition and tumour response.

Model	Treatment	Cancer	Main findings	Reference
Human (*N* = 55)	Chemoradiation (EBRT > 45 Gy/25 followed by intracavitary brachytherapy with concurrent systemic cisplatin)	Cervical cancer	Pre-RT. Enrichment in g_*Escherichia Shigella*, f_Enterobacteriaceae, and o_Enterobacteriales in faecal microbiota samples of long-term survivors (patients who had a follow-up of 2 years or more from baseline). Pre-RT. Enrichment in g_*Porphyromonas*, f_Porphyromonadaceae, and g_*Dialister* in faecal microbiota samples of short-term survivors (patients who had a follow-up of 1 year or less from baseline). Pre-RT. A high relative abundance of g_*Veillonella* is associated with improved median overall survival (non-significant).	[8]
Human (responder = 7, non-responder = 38)	Concurrent chemoradiation (5-FU/leucovorin or oral capecitabine; 50.0 Gy/25 50.4 Gy/28 54.0 Gy/30)	Rectal cancer	Pre-RT. Higher abundance of o_Bacteroidales (f_Bacteroidaceae, f_Rikenellaceae, g_*Bacteroides*) in patients with non-CR than those with CR. Pre-RT. S*_Duodenibacillus massiliensis* was associated with improved CR rate.	[73]
Mouse	Irradiation (16 Gy)	Breast cancer and melanoma	Pre-RT. An antibiotic cocktail of ampicillin, imipenem, cilastatin, and vancomycin before RT reduced RT efficacy.	[16]
Mouse	Irradiation (20 Gy)	Colon adenocarcinoma	Pre-RT. Oral vancomycin (mostly targeting Gram-positive bacteria) increased IR efficacy with decreased f_Lachnospiraceae.	[15]
Mouse	Irradiation (6 Gy)	Bladder cancer	Pre-RT and post-RT. Higher abundance of s_*Bacteroides acidifaciens* in responders to irradiation compared to non-responders within the soluble high-fibre diet group.	[21]
Mouse	Irradiation (21 Gy)	Melanoma, lung/cervical cancer	Pre-RT. Depletion of Gram-positive bacteria (two major families of SCFA-producing c_Clostridia: f_Ruminococcaceae and f_Lachnospiraceae) by vancomycin improved anti-tumour effects of radiotherapy.	[14]

*5-FU* 5-fluorouracil, *EBRT* external beam radiation therapy, *Gy* Gray.

## The role of SCFAs in health and cancer

Short-chain fatty acids (SCFAs) are fermented from dietary fibres by the gut microbiota. The three most common SCFAs, namely, acetate (C2), propionate (C3), and butyrate (C4), are produced primarily by two phyla of bacteria; Firmicutes, which produce butyrate, and Bacteroidetes, which produce acetate and propionate [10]. They account for approximately 90 to 95 percent of all SCFAs produced, in a constant molar ratio of 3:1:1, and the minor SCFAs are isobutyric, valeric (C5), isovaleric and caproate acid (C6). Colonocytes absorb SCFAs following their production in the gut; most of the butyrate absorbed and metabolised as fuel. Propionate travels along the portal circulation to the liver, where it is taken up by hepatocytes for energy. Acetate cannot be oxidised in the liver, and so enters the systemic circulation, with the remaining unabsorbed SCFAs, to travel to other target tissues [74]. Butyrate maintains intestinal barrier integrity by increasing the expression of claudin, a tight junction protein [75], and stabilises hypoxia-inducible factor (HIF), thereby increasing the expression of its barrier-protective target genes [76].

### Mechanisms of action of SCFAs

**Fig. 2:**
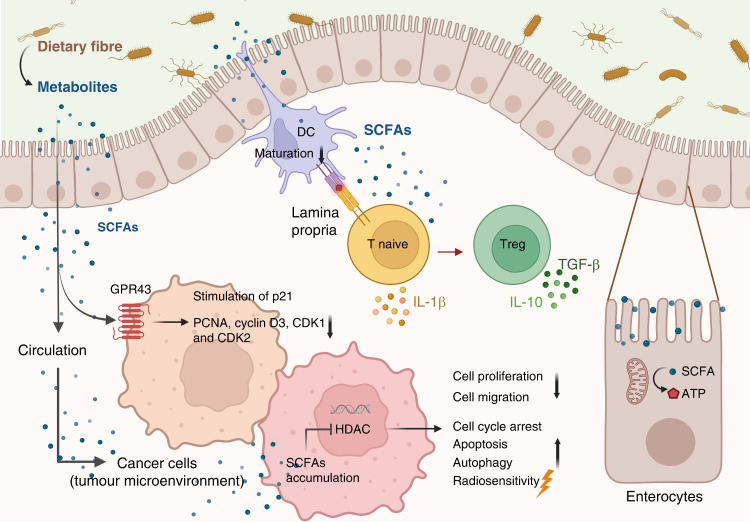
The influence of dietary fibre metabolites on signal transduction pathways in the gut. In colonocytes, butyrate acts as the major energy source. In the lamina propria, SCFAs suppress the maturation of dendritic cells but promote the development of Tregs. In the tumour microenvironment, SCFAs act on cancer cells via GPCR and HDAC inhibition (based on in vitro studies). SCFAs downregulate PCNA, cyclin D3, CDK1 and CDK2, and stimulate p21 expression via GPR43-dependent mechanisms. SCFAs also suppress cancer cells’ proliferation and migration, and promote cell cycle arrest, apoptosis, autophagy and radiosensitivity via inhibition of HDAC activity. Adapted from “Gut Microbial Environment with Peyer’s Patch”, by BioRender.com (2020). Retrieved from https://app.biorender.com/biorender-templates.

The effects of SCFAs are mediated via two major mechanisms, extracellular binding to their corresponding G-protein-coupled receptors (GPCRs), GPR41, GPR43, GPR109A (butyrate only), leading to a plethora of downstream signalling pathways, and acting intracellularly as class I and IIa HDAC inhibitors (Fig. 2). The GPR43 is expressed along the whole gastrointestinal tract, and propionate and acetate are the most potent and selective activators.

Butyrate was one of the first discovered histone deacetylase (HDAC) inhibitors [77], and has the strongest HDAC inhibitory activity of the SCFAs [78]. By allowing hyperacetylation of their corresponding histone proteins, the SCFAs increase the accessibility of transcription factors to the promoter regions of target genes, thereby modulating gene expression epigenetically [79]. SCFA receptors are expressed in the intestine, immune cells, adipose tissue, neurons, skeletal muscles and the heart. This widespread expression partly explains their involvement in various diseases [80]. Here, we will focus on their role in normal gut tissue and as anticancer treatment in terms of immune and non-immune effects (Fig. 2).

#### Effects of SCFAs on gut toxicity

The abundance of SCFA-producing bacteria from the phyla Firmicutes and Bacteroidetes is decreased in the gut microbiota of patients following radiotherapy and in those suffering from post-radiation inflammatory conditions [62, 81]. Topical sodium butyrate was shown to be effective in treating acute radiation proctitis in patients with pelvic malignancy after receiving radiotherapy [82]. Hence SCFAs may have a role in the alleviation or prevention of radiotherapy-induced toxicities, although the underlying mechanisms need to be determined.

#### Effects of SCFAs on gut immunity

SCFAs mediate communication between the gut microbiota and the mucosal immune system. For example, butyrate can inactivate nuclear factor kappa B (NF-κB) [83] and downregulate the production of tumour necrosis factor (TNF), decreasing inflammation, and it can decrease oxidative stress in the colon, improve gut barrier function, and protect against colon cancer and ulcerative colitis (an inflammatory bowel disease) [84, 85]. In vitro incubation of SCFAs with healthy human colon cells showed decrease in NF-κB activity, which has also correlated with improvement of colitis in mice in vivo [83].

SCFAs also impact the maturation of DCs which in turn stimulate regulatory T cells (Tregs). The microbiota has also an ongoing crosstalk with Tregs, inducing secretion of immunosuppressive cytokines [54]. Furusawa et al. reported a positive correlation between SCFA concentrations and colonic Tregs in mice fed a high-fibre diet (HFD), and found in vitro that butyrate, at physiological concentrations, significantly induced naive CD4^+^ T cells into Foxp3^+^ IL-10-producing Treg cells via HDACi activity. These results translated in vivo with a butyrate-containing diet ameliorating colitis induced by adoptive transfer of CD4^+^ CD45RBhi naive T cell into Rag^−/−^ mice, with a higher colonic Foxp3^+^ Treg­ population compared to the control group [86]. Peripheral T­reg induction and proliferation as well as de novo generation, at sites other than colon, have also been demonstrated by feeding propionate- and butyrate-containing water to mice treated with broad-spectrum antibiotics [87].

The current literature is inconsistent regarding SCFAs’ immune effects. Some studies have shown that higher levels of SCFAs stimulate pro-inflammatory cytokine-producing T cells in contrast to their generally agreed anti-inflammatory role [88, 89]. This agrees with studies which found an increased abundance of SCFAs in patients with post-radiation RE and diarrhoea [90].

#### Effects of other metabolites on gut toxicities and immunity

The gut microbiota, *Lachnospiraceae* and *Enterococcaceae* and SCFAs, especially propionate, have been shown to mitigate radiation-induced intestinal damage and the radioprotection was also associated with two tryptophan pathway metabolites, namely, 1H-indole-3-carboxaldehyde(I3A) and kynurenic acid (KYNA) [11]. Xiao et al. also demonstrated that gut microbiota-derived indole 3-propionic acid reduced gastrointestinal injury after radiation exposure. It attenuated local and systemic inflammatory levels, such as IL-6 and TNFɑ in the small intestine and peripheral blood, and mitigated radiation-induced colonic shortening [91]. In addition, valeric acid improved intestinal epithelial integrity and the recovery of gut microbiota after total abdominal irradiation [13]. Urolithin A, produced from the transformation of ellagitannins by gut microbiota, decreased the radiation-induced enterocyte apoptosis in the small intestine and restored gut microbiota changes after total body irradiation [12].

#### Effects of SCFAs on cancer cells

An interesting inconsistency for SCFAs in the literature is the “butyrate paradox”, in which butyrate stimulates cell proliferation of healthy colonocytes in vitro and in vivo but demonstrates opposing anticancer effects in cancer cell lines [92]. Donohoe et al. were the first to explain the paradox via the Warburg effect. In normal colonocytes, butyrate is rapidly metabolised via β-oxidation for fuel, and little remains in the cytoplasm. However, in cancerous cells, as a survival adaptation, glucose is preferentially used as fuel over SCFA, so the spared cytoplasmic butyrate can travel into the nucleus where it acts as an HDACi, upregulating the expression of genes involved in anti-tumour activities [93]. This property of butyrate makes it an attractive candidate as a potential tumour radiosensitiser which will be further discussed.

All three major SCFAs have demonstrated anticancer effects, but butyrate’s role has been studied most extensively due to its more potent activity as an HDACi compared to propionate and acetate [94]. For example, propionate induced apoptosis in lung cancer cells [95], and butyrate and propionate suppressed the proliferation of breast cancer cells [96]. Acetate, propionate, and butyrate increased histone deacetylase inhibition and radiosensitivity in human bladder cancer cells [97].

Despite these benefits of SCFAs, physiological concentrations of SCFAs in human plasma are low. In a study where healthy subjects were put on an HFD, taking 35 g of dietary fibre per day for 5 days, despite significantly increased SCFA concentrations in the HFD group, there were no changes to concentrations of anti-inflammatory cytokines and the number of circulating Tregs [98].

#### Effects of SCFAs on the tumour microenvironment

SCFAs can boost cellular metabolism and memory potential in CD8^+^ T cells [99], which may mean that SCFAs can promote a favourable tumour microenvironment in bladder cancer, in which T-cell inflamed tumours [100] with increased FOXP3^+^ Treg infiltration [101] have been associated with improved patient survival. Their anti-inflammatory properties also point them to a potential treatment for IR-induced RE.

## Modulating the gut microbiota to improve radiotherapy outcomes in pelvic cancers

The gut microbiota can be modulated by administering live microorganism (probiotics) or altering the fibre that they feed upon (prebiotics—see later) and such approaches are cheap and relatively safe. As diet has a major impact on gut health in terms of microbiota composition, diversity, and richness, dietary fibre manipulation could be a promising approach to target the gut microbiota effectively in a short period of time [102] to enhance RT efficacy or mitigating side effects. We hypothesise that high fibre positively influences tumour responses via fermentation of the high fibre and/or immunomodulation and reinforces the pre-RT gut microbiota to be more resistant to changes caused by IR. An alternative hypothesis is that it restores the gut microbiota during radiotherapy, thereby reducing the effects of the unfavourable IR-altered microbiota. Important questions need to be answered to determine the timing of dietary fibre supplements in RT patients to maximise the therapeutic ratio.

### Tumour radiosensitisation and modulation of the gut microbiota

Radiotherapy results in normal tissue toxicity and tumour cell death. IR kills cells by inducing lethal double-strand breaks (DSBs) in the genome. The cell detects this damage and initiates DNA repair mechanisms, both error-prone non-homologous end-joining (NHEJ) and accurate homologous recombination (HR), which rescue the cell from apoptosis. Molecules that can interfere with DNA repair or signalling processes can therefore act as radiosensitisers, increasing the number of DSBs lesions following IR and promoting apoptosis [103].

Butyrate acts on key players in DNA repair, namely, the DNA protein kinase catalytic subunit (DNA-PKcs), Ku70/Ku80 and ligase IV/XRCC4/XLF (Lig IV complex) [104]. A study utilising three-dimensional cultured organoids developed from intestinal stem cells from colorectal cancer (CRC) patients to mimic the native intestinal structure and environment, found that butyrate was the only SCFA to suppress the proliferation of cancerous organoids, and only radiosensitised tumour cell-derived organoids while sparing normal tissue organoids via the Warburg effect [105]. Furthermore, HDACi have been shown to radiosensitise tumours in vivo while sparing normal intestine from acute (panobinostat [106]) and late effects (romidepsin [107]). Therefore, it is rational to postulate that radiosensitisation might be achieved by increasing the population of SCFA-producing bacteria within the gut microbiota.

A study demonstrated that feeding a high soluble fibre (HSF) diet with 10% inulin to mice transplanted with human bladder cancer cells slowed tumour growth after irradiation compared to mice fed diets with lower fibre content. In the HSF group, an increased abundance of *Bacteroides acidifaciens* was found, which positively correlated with survival [97]. *B. acidifaciens* produces acetate, but faecal butyrate levels were found to be increased. Since acetate is needed for butyrate synthesis via the butyryl-CoA:acetate CoA transferase pathway [108], the putative explanation for this phenomenon is cross-feeding, where *B. acidifaciens* produces acetate to cross-feed other, butyrate-producing, bacteria in the gut microbiota [109]. The radiosensitising effect observed may possibly be attributed to increased SCFA production in the HSF diet-fed mice due to enriched *B. acidifaciens*. This finding opens an exciting avenue in which promising radiosensitisers like butyrate can be enriched in patients via the low-cost, non-toxic approach of dietary intervention. Such research is in its infancy; more robust in vivo data are required, followed by clinical trials, before such an approach can enter routine clinical practice.

There are relatively limited in vivo studies of gut microbiota and the efficacy of radiotherapy. Three studies all used an antibiotics-based approach to deplete specific groups of gut microbiota to change the RT efficacy and two studied cancers outside the gastrointestinal tract. Vancomycin, an antibiotic that kills Gram-positive bacteria in the gut, successfully enhanced radiotherapy efficacy via increasing dendritic cell antigen presentation [14, 15]. In contrast, an antibiotic cocktail of ampicillin, imipenem, cilastatin, and vancomycin reduced efficacy of radiotherapy with depletion of the commensal bacteria [16].

### Amelioration of radiation-induced toxicity

#### Probiotics

Probiotics are live microorganisms that, when administered in adequate amounts, confer health benefits to their host by altering the metabolic and nutritional function of the commensal microbiota [110]. They resist injury caused by gastric acid and bile to reach the large intestine intact. They can be obtained either from supplements or from specific foods prepared by bacterial fermentation. Probiotics, usually complex combinations of microorganisms, most commonly involve the *Lactobacillus* and *Bifidobacterium* bacterial genera, and have a specific mechanism of action [111].

Probiotics can modulate intestinal immune functions - decreasing pro-inflammatory cytokines, increasing secretory IgA and promoting tolerogenic cytokine profiles and regulatory pathways. They help reduce inflammation within the gut by optimising the epithelial barrier function, promoting cytoprotective responses and increasing mucin secretion. Moreover, probiotics limit possible harm by pathogenic bacteria: they decrease pathogen binding sites, reduce luminal pH and aid the production of anti-bacterial bacteriocins. However, little is currently known about the effects of probiotics on RT tumour responses.

The benefits of probiotics in reducing radiation-induced adverse effects of cervical cancer therapies within the gastrointestinal tract have been extensively studied. *L. acidophilus* LA-5 plus *B. animalis* subsp. *lactis* BB-12 were associated with significantly reduced use of loperamide, an antidiarrheal medication, among the probiotic patients, with a reduced incidence of grade 2 abdominal pain and episodes of abdominal pain in days after receiving radiotherapy [112]. In patients treated with chemotherapy and radiotherapy taking a mixture of probiotics and prebiotics over a 7-week period, faecal calprotectin was reduced as well as their frequency and intensity of vomiting [113]. Supplementation with the probiotic mixture VSL resulted in reduced daily bowel movements and a reduced incidence of radiation-induced diarrhoea [114].

In patients with gynaecological malignancies undergoing radiotherapy, fermented milk containing live *L. acidophilus* bacteria (and 6.5% lactulose as the probiotic substrate) reduced radiation-induced diarrhoea [115]. Moreover, patients supplemented with *L. rhamnosus* (Antibiophilus) had less radiation-induced gastrointestinal toxicity, with improved faecal consistency and fewer bowel movements [116].

#### Prebiotics

A prebiotic is “a non-digestible compound that, through its metabolism by microorganisms in the gut, modulates composition and activity of the gut microbiota, thus conferring a beneficial physiological effect on the host” [117]. Most prebiotics are classified as dietary fibres and are defined as substances fermented by intestinal microbiota that encourage specific intestinal bacteria growth and action. They must be resistant to gastric acid, hydrolysis by gastrointestinal enzymes and absorption across the epithelium so that they reach the large intestine intact. The gut microbiota and host exhibit a symbiotic relationship where gastrointestinal bacteria metabolise dietary polysaccharides, indigestible by human enzymes. This relationship makes the microbiota subject to modulation based on dietary intake (of specific prebiotics and fibre) since their growth and action is dependent on substrate availability [117].

Some recommendations advise a low-fibre diet while undergoing pelvic radiotherapy, but, in contrast, one randomised controlled trial (RCT) showed that a high-fibre diet may decrease GI toxicity and associated symptoms [118]. In patients with endometrial, cervical, colon, rectal or prostate cancer, prebiotics, partially hydrolysed guar gum (PHGG; natural water-soluble fibre source) reduced the frequency of diarrhoea following pelvic radiation treatment [119].

A murine study researching the effects of oat bran in reducing intestinal inflammation after pelvic irradiation reported similar findings and further suggested that a low-fibre diet may be harmful to patients [120]. A human RCT compared the effect of Metamucil (psyllium husk, a dietary fibre) in preventing diarrhoea related to pelvic radiotherapy [121]. Metamucil resulted in lowered severity of diarrhoea than placebo. An RCT studying the effect of fibre (inulin/fructooligosaccharides) compared to placebo in preventing RE in gynaecological cancer patients undergoing radiotherapy found that taking inulin generally improved stool consistency [122]. They had previously found that abdominal radiotherapy reduced *Lactobacillus* and *Bifidobacterium* counts but supplementation with an inulin and fructooligosaccharides mixture improved the recovery of both genera post-therapy [123]. However, there was no significant improvement in symptoms. One preparatory study recently reported plans for a large RCT investigating dietary fibre intake during pelvic radiotherapy [124], and, as available evidence continues to amass, this approach may soon impact clinical practice.

#### Faecal microbiota transplant

Faecal microbiota transplant (FMT), a procedure where donor faeces are mixed with saline solution before being transplanted into a patient (often through colonoscopy) to change their gut microbiota, has been used to treat various gastrointestinal conditions, including *Clostridiodes difficile* infection, showing to be effective [125–127]. NICE guidelines now support FMT as treatment for recurrent *C. diff* infections. It has also been investigated as a treatment for refractory immune checkpoint inhibitor-associated colitis (an immunotherapy side effect) [128], with one patient having a significantly increased prevalence of *Blautia* and *Bifidobacterium* species after FMT, which has been linked to decreased levels of intestinal inflammation [129]. Therefore, FMT may potentially be useful as a treatment for gastrointestinal toxicity caused by pelvic radiotherapy.

In a murine study, FMT post-radiation increased the survival rate of young mice [130]. However, when the experiment was replicated using older mice, their survival was not increased by FMT. Therefore, FMT may not solve the problem of post-radiation gastrointestinal toxicity in the elderly population. Evidence indicates the elderly population have an altered microbiota composition including reduced diversity, more Proteobacteria/other potentially pathological organisms, and fewer SCFA-producing species [131, 132].

A five-patient pilot study investigating FMT as a treatment for chronic RE caused by pelvic radiotherapy found that three patients responded to FMT, including a reduction in symptoms and physical improvement in mucosal injury on endoscopy [133]. Notably, symptoms recurred, possibly indicating a need for multiple FMT’s long-term. Likewise, a case report on chronic haemorrhagic radiation proctitis found that FMT relieved associated symptoms [134]. Despite these promising results, larger research studies are needed to test their applicability in the wider population.

## Conclusions and future perspectives

Research is now emerging into the influence of the gut microbiota on pelvic cancers, and the possibility of exploiting this in radiotherapy-based treatments, to improve both tumour control and side effects, is an exciting prospect. However, further studies are required to clarify the in-depth mechanisms, to provide evidence of the causal effects of the gut microbiota, the metabolome and immunity, and to explore the other potential dietary fibres or combinations to maximise the therapeutic ratio of radiotherapy.

Evidence is currently lacking regarding the involvement of the gut microbiota in late radiation-induced bowel toxicity; if modulation were possible, this could make a real impact on improving patient outcomes long-term.

Modulating the gut microbiota by dietary fibre manipulation may be particularly useful in the elderly, who cannot tolerate current chemoradiation schedules and whose microbiota composition is less favourable than that found in young patients. They therefore may have a gut microbiota with the most to gain in terms of increasing bacterial diversity, elimination of potentially pathogenic organisms and increasing SCFA-producing species. As evidence for dietary fibre manipulation is still relatively limited, more clinical studies with robust data are required before these strategies can be applied clinically.

In addition, the “baseline” that exists in the RT population varies hugely as intake of dietary fibre, microbiota profiles, production of SCFA and gut motility are highly individual and heterogenous. Further work should include clinical studies to better understand the baseline from which we are operating and the potential for improvement. Such work might help to identify those patients who would benefit most from these novel interventions.

## Supplementary information

Reproducibility check list

## Data Availability

Not applicable.
